# Phosphodiesterase-5 inhibitors for colon cancer chemoprevention

**DOI:** 10.18632/aging.101545

**Published:** 2018-09-05

**Authors:** Bianca N. Islam, Darren D. Browning

**Affiliations:** 1 Department of Biochemistry and Molecular Biology, Medical College of Georgia, Augusta University, Augusta, GA 30912, USA

**Keywords:** phosphodiesterase, colon, cGMP, cancer, prevention

Cancer of the colorectum is one of the most commonly diagnosed, and has a high mortality owing to the advanced stage at diagnosis. Chemoprevention is therefore a high priority for people at higher risk than the general population. While there are currently no drugs approved for this purpose, recent advances suggest that cyclic-guanosine monophosphate (cGMP) elevating agents could be effective. This second messenger has the well-established role of stimulating secretion in the small intestine, and its levels are tightly controlled by the activity of guanylyl cyclase C (GC-C) receptors that convert GTP into cGMP upon stimulation by the endogenous peptide hormones guanylin and uroguanylin [1]. Additional functions for cGMP have long been suspected because the cGMP synthesis machinery is also present in the colon where fluid reabsorption predominates. Genetic studies have shown that mice that are deficient in cGMP signaling components exhibit an intestinal epithelium that turns over more rapidly, exhibits barrier dysfunction, and is more susceptible to tumorigenesis. These observations clearly indicate an additional role for cGMP signaling in the regulation of intestinal homeostasis. Pharmacological studies in wild type mice confirmed this idea, as increasing cGMP using either GC-C agonists or phosphodiesterase 5 (PDE5) inhibitors increased epithelial barrier function and suppressed intestinal carcinogenesis.

The clinical utility of cGMP-elevating agents for colon cancer has long been an area of interest. Based on reports that guanylin expression was reduced in colon tumors compared to surrounding tissue, and that GC-C agonists inhibited colon cancer cell growth *in vitro*, it was originally thought that GC-C agonists could be an effective treatment for colon cancer patients. This idea was supported by work with the weak PDE5 inhibitor sulindac sulphone (exisulind) that killed colon cancer cells *in vitro*, and caused regression of colorectal polyps in human patients [2]. The idea that cGMP-elevating agents might prevent colon cancer arose from reports of the barrier-protective effects of cGMP, because inflammation is an established risk factor. Since the PDE5 inhibitor vardenafil was shown to suppress dextran-sulfate sodium (DSS)-induced inflammation in mice [3], it was reasoned that it might also prevent polyp formation in the azoxymethane (AOM)/DSS model of inflammatory carcinogenesis. Indeed, it was subsequently shown that the PDE5 inhibitor sildenafil suppressed polyp multiplicity in this mouse model by 50% [4]. An unexpected result from that landmark study was that polyp formation was similarly reduced when the sildenafil was withdrawn prior to DSS-treatment. This suggested that sildenafil primarily affected the initiation process, and that suppression of inflammation was not central to chemoprevention. This idea was further tested in the classical *Apc*^Min/+^ mouse model where polyp initiation occurs by somatic mutation leading to loss of heterozygosity at the *Apc* locus, and inflammation is not a central driver in the small intestine. Treatment of *Apc*^Min/+^ mice with either sildenafil or the GC-C agonist linaclotide equally suppressed polyp multiplicity by 50% [5].

Taken together, these studies demonstrate that increasing intestinal cGMP levels can reduce intestinal tumorigenesis in mice by targeting the early stages of tumor initiation. This latter point is supported by the fact that treatment with either sildenafil or linaclotide only affected the number of polyps per animal and did not affect the size or apoptotic index of polyps that formed in either preclinical model. While the reason for the lack of effect on initiated polyps warrants further investigation, these observations clearly do not support the utility of these drugs as a treatment for colon cancer patients. The prevention-effect of cGMP-elevating drugs is likely due to their ability to reduce the size of the proliferative compartment where tumor initiation occurs in the colon [6]. This effect of cGMP in healthy intestinal epithelium would reduce both the rate of spontaneous mutations as well as susceptibility to exogenous genotoxic stress, and would translate into reduced “risk” of tumor initiation in humans. While the extent to which PDE5 inhibitors will similarly affect intestinal homeostasis in humans is unknown, a recent report that used linaclotide in humans is encouraging [7]. The study found that the patients who responded to linaclotide with increased cGMP levels in the colon epithelium also exhibited reduced proliferation.

Linaclotide (and plecanatide) are synthetic GC-C agonists that were developed for the treatment of constipation by capitalizing on the pro-secretory role of cGMP. However, these drugs override endogenous autoregulatory processes, and diarrhea is a common side effect that could limit their utility for colon cancer chemoprevention [8]. PDE5 inhibitors are ideal for chemoprevention due to their low side-effect profile, and are routinely prescribed for the long term daily treatment of pulmonary arterial hypertension and benign prostate hyperplasia. Indeed, the preclinical studies described above truly validate PDE5 as a prevention target because they used the equivalent of a pediatric dose of sildenafil. The current state of affairs holds promise for people that are predisposed to developing colon cancer. In the near future they finally have the ability to reduce their risk by taking a small daily dose of a class of drugs with an extensive safety history.

## References

[1] Forte LR Jr. Pharmacol Ther. 2004; 104:137–62. 10.1016/j.pharmthera.2004.08.007 15518884

[2] Arber N, et al. Gut. 2006; 55:367–73. 10.1136/gut.2004.061432 16150858 PMC1856089

[3] Wang R, et al. Cell Death Differ. 2014; 21:427–37. 10.1038/cdd.2013.163 24270408 PMC3921591

[4] Islam BN, et al. Cancer Prev Res (Phila). 2017; 10:377–88. 10.1158/1940-6207.CAPR-17-0015 28468928 PMC5530733

[5] Sharman SK, et al. Cancer Prev Res (Phila). 2018; 11:81–92. 10.1158/1940-6207.CAPR-17-0267 29301746 PMC5811348

[6] Sharman SK, et al. PLoS One. 2017; 12:e0176673. 10.1371/journal.pone.0176673 28448580 PMC5407793

[7] Weinberg DS, et al. Cancer Prev Res (Phila). 2017; 10:345–54. 10.1158/1940-6207.CAPR-16-0286 28396341 PMC5758862

[8] Shah ED, et al. Am J Gastroenterol. 2018; 113:329–38. 10.1038/ajg.2017.495 29380823 PMC7213047

